# Episiotomy related morbidities measured using redness, edema, ecchymosis, discharge and apposition scale and numerical pain scale among primiparous women in Mulago National Referral Hospital, Kampala, Uganda

**DOI:** 10.11604/pamj.2020.36.347.25049

**Published:** 2020-08-26

**Authors:** Pebalo Francis Pebolo, Ajeani Judith, Kaye Kabonge Dan

**Affiliations:** 1 Department of Reproductive Health, Gulu University, Laroo, Uganda,; 2 Department of Obstetrics and Gynecology, Mulago National Referral Hospital, Kampala, Uganda,; 3 Department of Obstetrics and Gynecology, Makerere College of Health Sciences, Makerere University, Kampala, Uganda

**Keywords:** Episiotomy, REEDA scale, numerical pain scale, primiparity

## Abstract

**Introduction:**

episiotomy induced inflammatory signs like redness, edema, ecchymosis and pain may remain beyond the period of hospitalization and can be objectively measured using redness, edema, ecchymosis, discharge and apposition (REEDA) scale. Pain in the postpartum period is a common problem and can be measured using the numerical pain scale (NPS). Episiotomy is normally poorly executed and poorly repaired with little attention to the subtle pain-free scar. Postpartum perineal pain has been found to affect more people with episiotomy compared to spontaneous perineal tears or contusion in the first two weeks. This study was aimed at comparing NPS and REEDA scores in the first two weeks of postpartum among primiparous parturients with or without episiotomy in Mulago National Referral Hospital.

**Methods:**

a prospective cohort study conducted by recruiting primiparous women systematically on the first postnatal day and categorizing them into episiotomy and no episiotomy group. NPS and REEDA scale were taken at baseline and 2 weeks postpartum.

**Results:**

the mean total REEDA score for primiparous women among the episiotomy group was significantly higher both on day 1 and day 14 with p-values <0.0001 and <0.0001 respectively as well as the day 14 mean NPS p-value 0.001.

**Conclusion:**

episiotomy, a traumatic obstetric procedure, that heals slowly and with persistent perineal pain compare to spontaneous perineal contusion or tears.

## Introduction

Episiotomy is one of the most commonly practiced obstetric procedures done to enlarge the diameter of the vulvar outlet to facilitate the passage of the fetal head and prevent an uncontrolled tear of the perineal tissue in the second stage of labor [1]. It was initially described by Ould in 1741, to be applied in a mediolateral fashion in all births of nulliparous women to protect the fetal head from trauma and the pelvic floor from extreme lacerations [2], although the procedure is a potential danger for hematoma formation, perineal pain, infections, sexual dysfunctions and healing complication [3]. Episiotomy induced inflammatory signs such as redness, edema ecchymosis and pain occur in the first 24 hours and may remain beyond the period of hospitalization [4] and can be objectively measured using redness, edema, ecchymosis, discharge and apposition (REEDA) scale developed by Davidson [5]. This scale has been used in recent studies that have investigated interventions aimed at assessing perineal suture technique, postpartum perineal tear and the effect of laser irradiation on perineal pain [6]. Although assessing some of the parameters in REEDA scales such as ecchymosis and edema is unreliable in the first few days after perineal wounding, the scale is a reliable tool to assess perineal wound healing especially 7-10 days after delivery [7].

Pain in the postpartum period is a common problem and as in other conditions, measurement of pain has been a challenge. The numerical pain scale (NPS) for pain is a one-dimensional measure of pain intensity in adults including those with acute pain due to postpartum perineal trauma diseases [8]. The NPS is a segmented numeric version of the visual analog scale (VAS) in which a respondent selects a whole number (0-10 integers) that best reflects the intensity of their pain [9]. An episiotomy is normally poorly executed and poorly repaired with little attention to the subtle pain-free scar, postpartum perineal pain has been found to affect more people with episiotomy compared to spontaneous perineal tears or contusion in the first two weeks [1]. This study was therefore to compare perineal trauma-related morbidities and healing processes measured using NPS and REEDA scales in the first two weeks postpartum among primiparous women who had and those who had no episiotomy during delivery in Mulago National Referral Hospital.

## Methods

**Figure 1:**
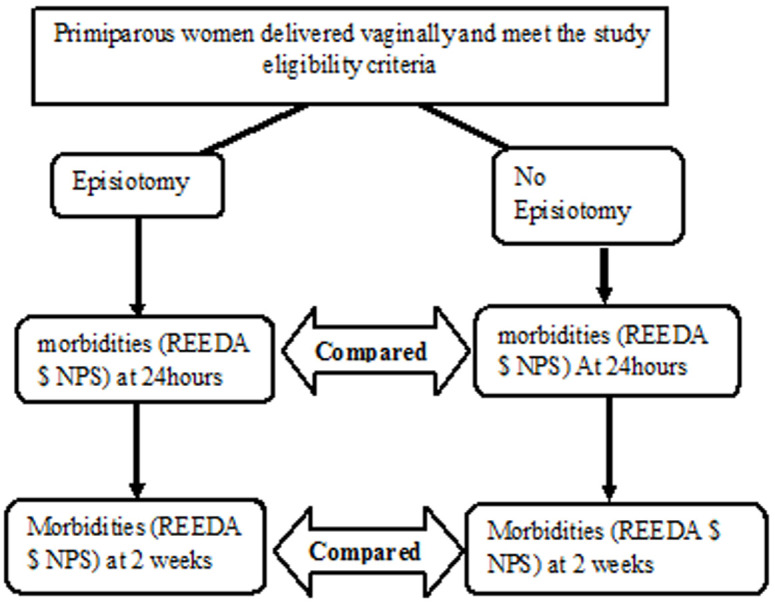
study design algorithm

A prospective cohort study conducted in Mulago National Referral Hospital labor suit, postnatal wards and clinic in Kampala, Uganda. The study was carried out in February and March 2018. Primiparous women who had spontaneous or assisted vaginal birth at 28 or more weeks of gestation and delivered from Mulago labor suits were recruited on the first postnatal day. A systematic random sampling method was used with a sample interval of two participants. At recruitment, they were categorized into those who had an episiotomy and those who did not have an episiotomy (Figure 1). NPS and REEDA scores were taken after informed consent and they were scheduled for review at two weeks postpartum. At the time of scheduling, their phone contacts and those of their next of kin were recorded and they were told that they should expect a reminder phone call about their scheduled visits. Those who did not have phones provided phone contacts of their husbands, mothers or someone they are staying with for easy follow-up. They underwent health education on wound care and postpartum Kegels exercise. Reminder phone calls were made 3 days prior to and a day before the appointment. Participants were reviewed at two weeks postpartum at the postnatal clinic and assessed for pain (as rated by the NPS) and perineal healing complication using the REEDA scale. Findings were explained to the participants and any complications diagnosed were managed accordingly or referred for specialist´s evaluation. Those who had not returned on the agreed date were reminded to come through a phone call. If they had not come after three reminders or at three weeks postpartum or confirmed of not coming back, they were considered as a lost to follow-ups. Approval to carry out this research was sought from: the Directorate of Obstetrics and Gynecology, Mulago National Referral Hospital and Makerere University, School of Medicine Research and Ethics Committee (SOMREC), reference #RECREF2018-023. The study was registered with the Uganda National Council of Science and Technology (UNCST).

### Tools for data collection

**REEDA scale:** the REEDA scale (Table 1) is a tool for assessing perineal healing that was primarily developed by Davidson. It includes five items related to the healing process: hyperemia, edema, ecchymosis, discharge and apposition of the wound edges (redness, edema, ecchymosis, discharge and apposition-REEDA) [5]. It can be used to assess all types of postpartum trauma including perineal trauma and has been used in recent studies that have investigated interventions aimed at assessing perineal suture techniques [6]. The scores range from 0 to 15, a lower score indicates better healing at the episiotomy site and higher score shows poor healing processes. The test reliability coefficient of the REEDA scale has been demonstrated to be r=0.70 [5].

**Table 1:** REEDA scale

Score	Redness	Edema	Ecchymosis	Discharge	Apposition
0	None	None	None	None	Closed
1	Within 0.25cm of incision/tear bilaterally	Perineal, <1cm from the incision/tear	Within 0.25cm bilaterally or 0.5cm unilaterally	Serous	Skin separation ≤3mm
2	Within 0.5 of Incision/tear bilaterally	Perineal and/or vulvar, 1-2cm from the incision/tear	Within 1.0 cm bilaterally or 0.5-2.0cm unilaterally	Serosanguinous	Skin and subcutaneous fat separated
3	Beyond 0.5cm of incision/tear bilaterally	Perineal and/or vulvar, >2cm from the incision/tear	>1cm bilaterally or >2cm unilaterally	Bloody, purulent	Skin, subcutaneous fat and fascial layer separation

Score: Redness________Edema________Echymosis______Discharge________Apposition_______Total score: ____________; interpretation of total score on REEDA scale; healed: 0; moderately healed: 1 to 5; mildly healed: 6 to 10; not healed: 11 to 15

**Numerical pain scale:** the NPS is a segmented numeric version of the visual analog scale (VAS) in which a respondent selects a whole number (0-10 integers) that best reflects the intensity of their pain [9]. The test reliability coefficient of NPS has been demonstrated to be r=0.62. All participants with an NPS of 5 or more were prescribed paracetamol for three days.

**Quality control:** before starting data collection, two research assistants were trained for 2 days on ethical guidelines, objectives and procedures of the study, the importance of informed consent and filling of the questionnaire by the principle investigator. Attention was paid on measurements of the REEDA scale using a tape measure. To better expose the perineal wound, an examination was performed when a woman was lying in lateral Sim´s position. Pretesting of the questionnaire was also done during the period of the training of research assistants where 20 mothers were included in the pretesting sessions. Raw data was checked at the end of each day, double entered and checked for consistencies.

**Data management and analysis:** data was entered into Epidata 3.1. Each record was assigned a unique identifier and names dropped to maintain participants' confidentiality. The quality of data was assessed by conducting consistency checks. An independent samples t-test was carried out to compare the mean score of NPS and REEDA among the women who had an episiotomy and those that did not, both at baseline and 14 days postnatal period. Multivariate logistics regression was used to tease out potential confounders such as duration of labor, gestational age, mother's age, marital status and religion among the many variables.

## Results

**Figure 2:**
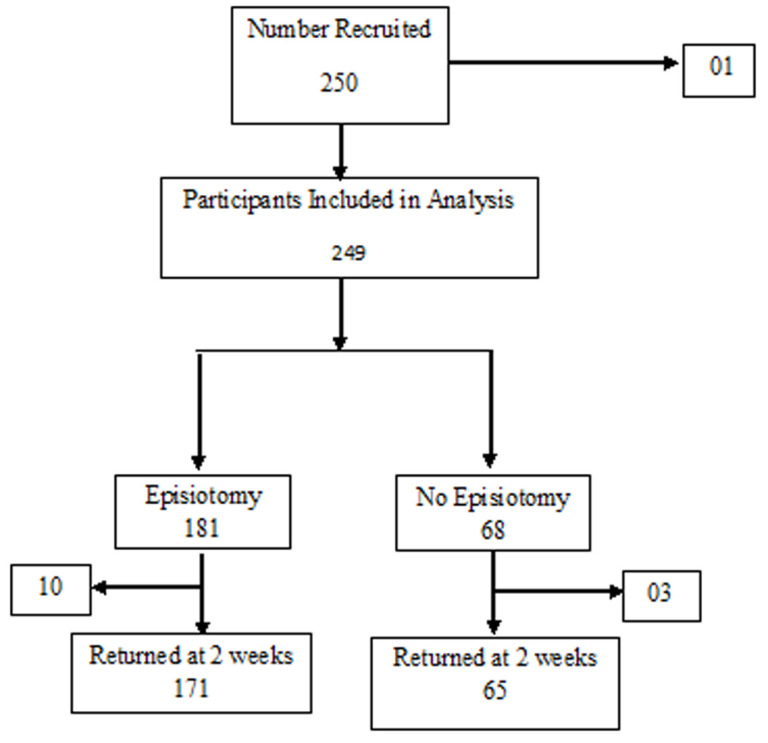
schema showing distribution of study participants

Two hundred and fifty participants were recruited and 249 included in the analysis, 181 had episiotomy, 68 had no episiotomy giving an episiotomy rate of 73% (CI 67-78) as reported elsewhere [10]. Thirteen people were lost to follow-up, 10 on the episiotomy arm and 3 on the no episiotomy arm (Figure 2). The average scores for REEDA, Individual morbidities in REEDA scale and NPS scale were compared among women who had episiotomy and those who did not. The comparison was done both for baseline and day 14 after the procedure. The results showed that the mean total REEDA score for women who had episiotomy was significantly higher than in those who did not have both on day 1 and day 14 with p-values <0.0001 and <0.0001 respectively. The mean NPS score for women who had an episiotomy and those who did not have were not significantly different on day 1 (baseline) p-value 0.541, but significantly different on day 14 with women who had episiotomy having higher mean NPS p-value<0.0001 (Table 2). The average scores for the REEDA scale were compared among women who had an episiotomy and those who did not have the procedure performed. The comparison was done both for baseline and day 14 after the procedure. The scores were adjusted for duration of labor, gestational age, mother's age, marital status and religion among the many variables considered in the final model. According to the models, there is a significant difference in average REEDA scores between mothers who had an episiotomy and those who did not (1.37 at baseline and 0.76 at day14) p-value <0.0001 and <0.0001 respectively. On day 14, there is a significant difference in average scores for younger mothers and older mothers. There is a significant difference between scores of women who took longer than 30 minutes in the second stage of labor and those who took less (Table 3). The NPS has also been adjusted for other factors associated with pain except for episiotomy. The pain at baseline is the same on average for patients that have episiotomy vs those who did not have. Participants who had longer labor experienced more pain than those with shorter labor. Participants with a higher level of education reported higher levels of pain on the scale compared with patients with lower education levels (Table 4). On day 14 however, patients with episiotomy had more pain than those without episiotomy after adjusting for all other confounders.

**Table 2:** bivariate comparison of morbidities as measured by REEDA and NPS among women with and without episiotomy, at baseline and day 14

	Day 1 (baseline)			Day 14		
Characteristics of morbidity	Frequency	Mean	p-value	Frequency	Mean	p-value
**Total REEDA score**						
**Mean total REEDA score**						
No episiotomy	68	0.78±0.14	^*^<0.0001	65	0.38±0.12	^*^<0.0001
Episiotomy	181	2.17±0.11		171	1.63±0.09	
**Individual morbidities in REEDA score**						
**Redness**						
No episiotomy	68	0.04±0.03	^*^<0.0001	65	0.06±0.03	^*^<0.0001
Episiotomy	181	0.41±0.04		171	0.45±0.04	
**Edema**						
No episiotomy	68	0.43±0.09	^*^<0.0001	65	0.08±0.03	^*^<0.0001
Episiotomy	181	0.98±0.06		171	0.46±0.04	
**Ecchymosis**						
No episiotomy	68	0	^*^0.0023	65	0.03±0.03	^*^0.0041
Episiotomy	181	0.19±0.04		171	0.19±0.03	
**Discharge**						
No episiotomy	68	0		65	0	0.1649
Episiotomy	181	0.04±0.02	0.149	171	0.03±0.01	
**Apposition**						
No episiotomy	68	0.31±0.06	^*^0.0051	65	0.20±0.06	^*^0.0002
Episiotomy	181	0.56±0.05		171	0.51±0.04	
**Pain score**						
**Mean NPS**						
No Episiotomy	40	1.65±0.18	0.541	65	0.12±0.04	^*^<0.0001
Episiotomy	165	1.76±0.07		171	0.54±0.06	

* Significant

**Table 3:** multivariate comparison of the difference in total REEDA scores of women with and without episiotomy at baseline and day 14

	REEDA Baseline				REEDA Day 14			
	Coef.	p-value	[95% Conf. Interval]		Coef.	p-value	[95% Conf. Interval]	
**Episiotomy**								
No	-				-			
Yes	1.37	<0.0001^*^	0.93	1.81	0.76	<0.0001^*^	0.43	1.09
**Labor duration**								
<30mins	-				-			
30-60mins	-0.09	0.675	-0.53	0.34	0.93	<0.0001^*^	0.61	1.25
>60mins	0.18	0.552	-0.43	0.80	0.83	<0.0001^*^	0.37	1.28
**Gestational age**								
<37	-				-			
≥37	-0.09	0.756	-0.66	0.48	-0.28	0.197	-0.70	0.15
**Age**								
<18	-				-			
18-22	0.27	0.545	-0.60	1.14	1.06	<0.0001^*^	0.43	1.70
>22	0.06	0.888	-0.83	0.96	0.63	0.059	-0.02	1.28
**Marital status**								
Married	-				-			
Single	0.02	0.940	-0.56	0.60	0.27	0.207	-0.15	0.70
Separated	0.92	0.202	-0.50	2.33	0.55	0.286	-0.47	1.58
**Religion**								
Christian	-				-			
Muslims	0.07	0.795	-0.45	0.59	-0.28	0.153	-0.66	0.10

* Significant

**Table 4:** multivariate comparison of the difference in NPS scores of women with and without episiotomy at baseline and day 14

	NPS at Baseline				NPS at Day 14			
	Coef.	p-value	[95% Conf. Interval]		Coef.	p-value	[95% Conf. Interval]	
**Episiotomy**								
No								
Yes		0.058	-0.01	0.69	0.35	0.001^*^	0.14	0.56
Duration of labor stage	0.34							
**<30mins**								
30-60mins	-0.53	0.001	-0.84	-0.21	0.07	0.523	-0.14	0.28
>60mins	-0.41	0.063	-0.85	0.02	0.09	0.567	-0.22	0.39
**Birth weight**								
<2.5								
2.5-3.5	0.15	0.702	-0.60	0.89	-0.10	0.657	-0.54	0.34
>3.5	0.66	0.110	-0.15	1.47	-0.01	0.982	-0.50	0.49
**Education level**								
1								
2	-0.14	0.366	-0.46	0.17	-0.02	0.855	-0.23	0.19
3	0.62	0.018	0.11	1.14	-0.08	0.659	-0.45	0.28
**Age category**								
<18								
18-22	0.08	0.845	-0.70	0.86	0.36	0.115	-0.09	0.82
>22	0.02	0.951	-0.76	0.80	0.31	0.194	-0.16	0.78
**Marital status**								
1								
2	-0.14	0.545	-0.59	0.31	0.03	0.847	-0.26	0.31
3	0.39	0.408	-0.54	1.32	0.44	0.198	-0.23	1.11

^*^Significant

## Discussion

**Morbidity associated with episiotomy:** this study aimed at comparing the morbidity as measured by REEDA scale and NPS in the first two postnatal weeks among primiparous women who had vaginal births in Mulago National Referral Hospital. The results revealed a significantly higher average REEDA score both on the first postnatal day and on day 14 among those with episiotomy compared to those with no episiotomy after adjusting for confounders. Although, the first postnatal average NPS was similar for both episiotomy and no episiotomy group, the day 14 average NPS scale was significantly higher in the episiotomy group after adjusting for confounders. The only episiotomy type practiced in this study was mediolateral of which 3 (1.7%) had episiotomy wound extension to at least a third-degree perineal tear. Also, of the 68 mothers not done episiotomy, 31 (45.6%) had intact perineum meanwhile 37 (54.4%) sustained perineal tear with 81.1% (30) sustaining first degree 18.9% (7) second-degree perineal tears and all these were observed in the posterior median perineal raphe. There were no cases of third- or fourth-degree perineal tears among the primiparous mothers not done episiotomy compared to the three 3 (1.7%) cases among those whose episiotomy wound extended to at least a third-degree perineal tear. Medio-lateral episiotomy has been adopted in most parts of the world except in the United States of America because of its benefits of reducing the risk of third- or fourth-degree perineal tear extension, but its shown to be associated with increased postpartum pain, difficulty in repair and poor wound healing [11].

This is because it normally cuts across tissue planes causing more trauma compared to spontaneous perineal tears along tissue planes. This study has given evidence contrary to the role of episiotomy being protective for third- or fourth-degree perineal tears as seen in a study done by [1] although it agrees with a narrative review that associated episiotomy and perineal laceration [12]. Inflammatory signs such as redness, edema ecchymosis and pain occur in the first 24 hours and may remain beyond the period of hospitalization [7]. The REEDA scale is a tool for assessing perineal trauma/healing that was primarily developed by Davidson [5]. In this study, it was found out that there were statistically significant differences in the mean total REEDA score between those who had an episiotomy and those who did not both on day 1 (baseline) and day 14 postpartum (p-values <0.0001 and <0.0001 respectively) after adjusting for confounders. This shows that episiotomy is associated with a higher mean total REEDA score both on the first postpartum day and as well as two weeks post-delivery compared to those women who had no episiotomy done. Since high REEDA score denotes severe tissue injury or poor healing process, episiotomy is, therefore, can be seen as more of a traumatic procedure that heals slowly or poorly when compared to those with no episiotomy but with resultant spontaneous perineal tears or tissue injuries.

These findings agree with a study done by Raina *et al*. 2016 that shows that higher REEDA scale is associated with episiotomy [13]. Comparison of the different REEDA score values showed that on the first postpartum day, an episiotomy is more associated with a higher mean score of redness, edema, ecchymosis and apposition with p-values of <0.0001, <0.0001, 0.002 and 0.005 respectively compared to those with no episiotomy. Very few mothers had discharge on the first postpartum day. The rate of gapping at two weeks postpartum was particularly higher among those done episiotomy 80/171 (47%) compared to those not done episiotomy 12/34 (35%). An explanation in the differences can be derived from the fact that most of the spontaneous perineal tears were of first degree compared to episiotomy which is a second-degree tear and up to 45.5% of those not done episiotomy had intact perineum. The differences can also be attributed to the fact that spontaneous perineal tear occurs normally along the natural tissue planes and it´s easier to repair compared to episiotomy. Studies have shown that mediolateral episiotomy is associated with difficulties in repair [11] and it is not surprising that since the only episiotomy done in this study was medio-lateral, the high rate of gapping can be attributed to the difficulty in episiotomy repair.

Pain during the immediate postpartum period is a common finding and its severity depends on the extent of tissue damage and the participant's pain threshold. In this study, the mean NPS was statistically significant on day 14 compared as opposed to day 1 postpartum, p-values 0.001 and 0.058 respectively after adjusting for confounders. This implies that the pain in episiotomy is more likely to persist for at least two weeks as episiotomy is more traumatic compared to the pain in those mothers who had no episiotomy. This supports the earlier claim that cutting across tissue planes is associated with more pain compared to spontaneous tear that normally follows the natural tissue planes as reported by [3,14,15] and that episiotomy is a painful policy [1]. A metanalysis done by [12], found out that episiotomy is associated with increased incidence and severity of postpartum perineal pain. The limitations in our study can arise from the fact that measurements for ecchymosis and redness in the REEDA scale may post some difficulties in dark skin. This can also cause inter-observer variability. Nevertheless, this limitation has been minimized by having the research assistants trained for two days before data collection.

## Conclusion

An episiotomy is, therefore, a traumatic procedure that heals slowly with pain that lasts longer and it should be practiced at a restricted rate of not more than 10% to reduce morbidities. A scheduled postnatal review must be done at two weeks postpartum especially among those with episiotomies or at least a second-degree spontaneous tear to identify and manage complications.

### What is known about this topic

- Prevalence of episiotomy and primi gravidae being a risk factor;
- Episiotomy being a risk factor for third degree perineal tear;
- Use of REEDA scale and NPS in measurement of episiotomy related morbidities and healing processes.

### What this study adds

- Relevance of two weeks scheduled postnatal review for mothers with episiotomy;
- Episiotomy related pains are more likely to persist for at least two weeks;
- Since episiotomy is associated with significant morbidity, it should be practice restrictively.
